# Estimating the Effect of Elagolix Treatment for Endometriosis on Postmenopausal Bone Outcomes: A Model Bridging Phase III Trials to an Older Real‐World Population

**DOI:** 10.1002/jbm4.10401

**Published:** 2020-11-07

**Authors:** Ryan D Kilpatrick, Stephanie E Chiuve, William D Leslie, Lani R Wegrzyn, Wei Gao, Hongbo Yang, Ahmed M Soliman, Michael C Snabes, Sarah Koenigsberg, Jia Zhong, Cheryl Xiang, Nelson B Watts

**Affiliations:** ^1^ Global Epidemiology, Pharmacovigilance & Patient Safety, AbbVie, Inc. Chicago IL USA; ^2^ University of Manitoba Winnipeg Manitoba Canada; ^3^ Analysis Group, Inc. Boston MA USA; ^4^ Health Economics and Outcomes Research, AbbVie, Inc. Chicago IL USA; ^5^ Clinical Development, Global Pharmaceutical Research and Development, AbbVie, Inc Chicago IL USA; ^6^ Mercy Health Osteoporosis and Bone Health Services Cincinnati OH USA

**Keywords:** BONE MINERAL DENSITY, ELAGOLIX, ENDOMETRIOSIS, FRACTURE RISK

## Abstract

Elagolix, a gonadotrophin‐releasing hormone antagonist, is used in premenopausal women with endometriosis. There is a risk of bone loss with elagolix, but the long‐term effects of BMD loss later in life cannot be directly assessed and has not been quantified. To address this gap in knowledge, this study indirectly estimated the impact of elagolix on postmenopausal fracture risk. BMD change in premenopausal women with endometriosis treated with elagolix was modeled from the phase III program data (elagolix group) and used to simulate treatment effects on (fracture risk assessment tool estimated) 10‐year risks of hip and major osteoporotic fracture in women ages 50 to 79 years from the 2005–2010 National Health and Nutrition Examination Survey (NHANES; *N* = 2303). Change in the proportion of women reaching risk‐based antiosteoporotic treatment thresholds was also estimated. For elagolix versus NHANES, median 10‐year risk of major osteoporotic fracture was 4.73% versus 4.70% in women ages 50 to 59 years, 7.03% versus 6.97% in women ages 60 to 69 years, and 10.83% versus 10.68% in women ages 70 to 79 years. Median 10‐year risk of hip fracture in these same groups was 0.19% versus 0.18% for women ages 50 to 59 years, 0.51% versus 0.49% for women 60 to 69 years, and 2.22% versus 2.14% for women 70 to 79 years. The proportion of women reaching risk‐based antiosteoporotic treatment thresholds caused by elagolix 150 mg daily for 12 months was 0.36% higher at age 50 to 59 years, 0.23% at age 60 to 69 years, and 1.79% at age 70 to 79 years. The number needed to harm was 643 for one additional hip fracture and 454 for one additional major osteoporotic fracture. Results were similar for elagolix 200 mg twice a day for 3 months. In the modeled scenarios, elagolix had minimal impact on long‐term risk of fracture and reaching risk‐based treatment thresholds. © 2020 The Authors. *JBMR Plus* published by Wiley Periodicals, Inc. on behalf of American Society for Bone and Mineral Research © 2020 The Authors. *JBMR Plus* published by Wiley Periodicals LLC on behalf of American Society for Bone and Mineral Research.

## Introduction

Endometriosis is a potentially disabling, long‐term, estrogen‐dependent gynecological disorder characterized by growth of endometrial tissue outside of the uterus.^(^ ^1^ ^)^ Approximately 1 in 10 women of reproductive age in the United States suffer from endometriosis.^(^ ^2^, ^3^ ^)^ Of these, 60% experience significant chronic pain, including nonmenstrual pelvic pain, dysmenorrhea, and dyspareunia, which can worsen over time.^(^ ^4^, ^5^, ^6^, ^7^ ^)^ The disease is also associated with an increased prevalence of depression, reduction in sexual satisfaction, disrupted personal relations, and loss of work leading to substantial economic cost.^(^ ^8^, ^9^ ^)^

Several treatment options have been available for this patient population.^(^ ^3^, ^5^, ^10^ ^)^ First‐line medications include pain relievers such as nonsteroidal anti‐inflammatory drugs, and estrogen/progestin‐combined contraceptives or progestin‐only contraceptives.^(^ ^11^, ^12^, ^13^ ^)^ Surgical procedures, such as laparoscopy to remove endometriomas, can ameliorate symptoms in some women; however, pain symptoms often recur.^(^ ^14^ ^)^ Medical therapies, therefore, are the mainstay option for long‐term management.^(^ ^13^, ^15^ ^)^ For patients with incomplete pain resolution after using first‐line therapies, treatment with a second‐line hormonal therapy such as a gonadotropin‐releasing hormone receptor (GnRH) agonist or antagonist may be initiated.^(^ ^12^ ^)^ GnRH agonists first stimulate, then inhibit ovarian‐stimulating hormones to postmenopausal levels, reducing endometriosis associated pain, but leading to side effects that limit tolerability and the duration of use.^(^ ^5^ ^)^ Elagolix (brand name: Orilissa; AbbVie, North Chicago, IL, USA), an oral GnRH antagonist, was approved by the US Food and Drug Administration (FDA) in July of 2018.^(^ ^16^, ^17^ ^)^ Elagolix enables a dose‐dependent reduction in estrogen, and has demonstrated efficacy in alleviating moderate‐to‐severe endometriosis‐related pain with long‐term sustained effects.^(^ ^16^, ^18^, ^19^ ^)^ As a GnRH antagonist, elagolix may also reduce BMD through its estrogen‐reducing mechanism.

Low BMD is an important contribution to increased fracture risk and osteoporosis, which is associated with a substantial economic and societal burden.^(^ ^20^, ^21^ ^)^ In 2005, total costs attributed to fractures were estimated to be $19 billion, with a projected increase of >50% in annual fractures and their associated costs by 2025 among those aged 65 to 74 years.^(^ ^21^ ^)^ Although the prevalence of reduced BMD and the risk factors associated with bone loss have been widely studied in postmenopausal women, there is limited data available for premenopausal women, who would not ordinarily undergo regular DXA screening for BMD measurement.^(^ ^22^ ^)^ Data on the impact of premenopausal BMD reduction on long‐term postmenopausal fracture risk are also largely unavailable, particularly of the magnitude seen in the elagolix development program. Acknowledging this evidence gap, in 2017, Binkley et al. published the results of a simulation exercise to understand how therapies that affect BMD in premenopausal women may impact the time to reach guideline‐based treatment thresholds for osteoporosis.^(^ ^23^ ^)^ Although that study did not focus on any specific therapies, it provided a framework for use if relevant data were available and applied. To that end, the goal of this study was to utilize the data from the elagolix development program and the framework of Binkley et al. to estimate the impact of premenopausal changes in BMD in women treated with elagolix on long‐term, postmenopausal risk of major osteoporotic and hip fracture and the proportion of women who reached the threshold to initiate antiosteoporosis treatment at various ages.

## Materials and Methods

### Overview

In this study, premenopausal reduction and recovery in BMD caused by elagolix treatment was estimated using patient‐level data from the phase III clinical trials of elagolix. These trial‐derived estimates were then applied to observed postmenopausal BMD data from the 2005–2010 National Health and Nutrition Examination Survey (NHANES) to simulate postmenopausal BMD for a hypothetical cohort of endometriosis patients who received elagolix treatment.^(^ ^24^ ^)^ Both the model and simulation assumed that patients were not receiving antiosteoporotic medications that could reverse BMD loss.

In NHANES, BMD was measured via Hologic QDR 4500A fan‐beam dual‐energy X‐ray absorptiometers (Hologic, Inc., Marlborough, MA, USA). The standardized protocol for NHANES has been published previously^(^ ^25^ ^)^; all other data were collected through interview questionnaires and standardized physical examinations performed by trained medical professionals. NHANES data were previously collected with the approval of the National Center for Health Statistics Research Ethics Review Board and anonymized before release to the public. All participants provided informed consent prior to participation.

The FRAX (fracture risk assessment tool) Desktop Multi‐Patient Entry (version 4.0) was used to estimate the 10‐year risks of major osteoporotic fracture and hip fracture. Race‐specific FRAX algorithms for the United States were used. The analysis also estimated the proportion of women who reached the thresholds to initiate antiosteoporosis treatment per National Osteoporosis Foundation guidelines based on 10‐year risks of major osteoporotic fracture and hip fracture.

### Modeling approach to estimate elagolix‐induced premenopausal reduction and recovery in BMD

The on‐treatment reduction and the posttreatment recovery in BMD in women exposed to elagolix was based on clinical trial data from EM‐I (NCT0162058), EM‐II (NCT01931670), and their extension studies (EM‐III: NCT02143713 and EM‐IV: NCT01760954). EM‐I and EM‐II were two double‐blind, randomized, placebo controlled phase III trials that evaluated the efficacy and safety of elagolix in women ages 18 to 49 years with surgically diagnosed endometriosis who experienced moderate or severe endometriosis‐associated pain.^(^ ^16^ ^)^ Patients in both trials were randomized into one of three parallel dose groups in a 3:2:2 ratio to receive either placebo, elagolix 150 mg once daily (QD), or elagolix 200 mg twice daily (BID) for 6 months. After completion of EM‐I and EM‐II, qualified patients entered extension studies (EM‐III and EM‐IV) that investigated treatment outcomes following an additional 6 months of treatment.^(^ ^19^ ^)^ In EM‐III and EM‐IV, patients on active treatment continued to receive the same dose of elagolix, whereas patients administered placebo initially were randomized into one of the two active dose groups in a 1:1 ratio. The extension trials included a posttreatment follow‐up period of at least 6 months (ie, recovery period). However, the recovery data from EM‐III (the extension study of EM‐I) were only collected among women who had >8% decrease in BMD. Therefore, to minimize bias based on differential data collection, only the recovery data from EM‐IV (the extension study of EM‐II) were used in the analysis.^(^ ^19^ ^)^

The analysis was conducted on a randomly selected sample of 85% of patients from the pooled population of the pivotal trials. A linear cross‐sectional regression model was fitted using the baseline data from the elagolix and placebo arms of the EM‐I and EM‐II trials to identify the demographics and clinical characteristics associated with femoral neck BMD. Stepwise regression with Bayesian information criterion (BIC) as the selection criterion was used for variable selection.^(^ ^26^ ^)^ Then, a linear mixed effects model was fitted using the longitudinal data from the clinical trials to estimate the effects of the elagolix dose, elagolix treatment duration, and recovery duration on femoral neck BMD, adjusting for the demographic and clinical characteristics identified in the prior cross‐sectional model.^(^ ^27^ ^)^ The addition of quadratic terms and interactions between treatment‐related factors (elagolix treatment duration, dosing, recovery period) and patient characteristics did not improve model performance. These trial‐derived estimates for on‐treatment reduction and posttreatment recovery in BMD were used in the simulation described below.

### Simulation approach to estimate the effect of elagolix on postmenopausal bone health

To assess the long‐term effect of bone loss caused by elagolix, postmenopausal BMD was simulated for a group of hypothetical women aged 50 to 79 years who had received elagolix treatment prior to menopause (ie, elagolix‐treated group). Specifically, for each woman, elagolix‐exposed BMD was simulated by applying the modeled on‐treatment reduction and posttreatment recovery in BMD from trial data to each observed BMD in the 2005–2010 NHANES data. FDA‐approved treatment durations are up to 24 months for 150‐mg elagolix QD and up to 6 months for 200‐mg elagolix BID.^(^ ^17^ ^)^ Two scenarios were simulated. In the first scenario, all patients received 150 mg of elagolix QD for 12 months. In the second scenario, all patients received 200 mg BID of elagolix BID for 3 months. These 12‐month (150 mg QD) and 3‐month (200 mg BID) durations were chosen to reflect a realistic population‐level average treatment duration in the real‐world. In both scenarios, all patients were assumed to have 6 months of posttreatment recovery. BMD in a simulated elagolix‐exposed NHANES cohort was compared with the observed BMD in the NHANES sample, which was used as a referent group for BMD in the absence of elagolix treatment.

FRAX was used to estimate the 10‐year risk of osteoporotic or hip fracture for the simulated elagolix‐treated group and the referent group. FRAX is a previously validated tool that accounts for BMD and other fracture risk factors to predict long‐term osteoporotic and hip fracture risks.^(^ ^28^ ^)^ Patient‐level fracture risk factors required by FRAX were assumed to be the same between the elagolix‐treated group and the referent group and were extracted from the observed values in the NHANES data. The proportion of patients recommended to initiate antiosteoporosis treatment within the elagolix‐treated group and the referent group was calculated using the following thresholds: 10‐year probability of hip fracture ≥3% and 10‐year probability of major osteoporotic fracture ≥20%.^(^ ^29^ ^)^

The differences in the fracture risks and the proportion of patients who reached the thresholds to initiate antiosteoporosis treatment were calculated between the simulated elagolix‐treated group and the referent group. The analysis was conducted, separately, for the following age strata: (i) 50 to 59 years, (ii) 60 to 69 years, and (iii) 70 to 79 years. Subgroup analyses were conducted and stratified by percentiles of observed BMD in the absence of elagolix treatment (ie, <25th percentile, 25th to 75th percentile, >75th percentile). Number needed to harm (NNH) was calculated for both hip fracture and major osteoporotic fracture in both dosing groups.

Two sensitivity analyses were conducted. One explored the impact of elagolix on BMD among a population of patients in which 50% were treated with 150‐mg QD elagolix for 12 months and 50% were treated with 200‐mg BID elagolix for 3 months. An additional sensitivity analysis was conducted to determine the effect of rheumatoid arthritis prevalence on long‐term risks of fracture.

All analyses, except for the FRAX calculation, were conducted in SAS 9.4 (SAS Institute Inc, Cary, NC, USA).

## Results

### Elagolix‐induced premenopausal BMD reduction and recovery

Data from 999 study patients with nonmissing baseline BMD were used to identify patient characteristics associated with premenopausal femoral neck BMD levels in the cross‐sectional model. The identified patient characteristics included age, weight, race, use of genitourinary system medication, and sex hormones, blood diseases (eg, anemia, other blood diseases, no blood diseases), and ovary lesion. A total of 764 patients with 2226 observations were used to build the longitudinal mixed‐effects model. The estimated effect sizes of elagolix doses, treatment duration, and recovery duration that were used in the simulation of femoral neck BMD for hypothetical patients treated with elagolix are provided in Table 1.

**Table 1:** Elagolix‐Induced Reduction and Recovery in BMD Estimated by the Longitudinal Mixed Effects Model

	Estimate	SE	*P* value
Treatment duration (year)	−0.0051	0.0024	0.035
Treatment duration (year) × recovery duration (year)	−0.0003	0.0039	0.940
Treatment duration (year) × 200 mg BID (versus 150 mg QD)	−0.0188	0.0036	< 0.001
Treatment duration (year) × recovery duration (year) × 200 mg BID (versus 150 mg QD)	0.0086	0.0052	0.098

The model was adjusted for age, weight, race (white versus non‐white), use of genitourinary system medication and sex hormones as medication, anemia (versus no blood diseases), other blood diseases (versus no blood diseases), and ovary lesion.

BID = Twice daily; QD = once daily.

### Patient characteristics in the simulation study

A total of 2303 women from the 2005–2010 NHANES database were included in the analysis (Table 2). The mean age was 63 years (SD 8) and 58.1% were white. The mean BMI was 29.1 (SD 5.99), mean BMD *T*‐score was −0.96 (SD 1.12), 3.6% had evidence of prior fracture, and 21.7% had a parental history of fracture or osteoporosis. Baseline BMD among patients in the elagolix program was similar to baseline BMD among the NHANES population of the same age group.

**Table 2:** Patient Characteristics in the Simulation Study, All Patients, and Patients Who Met the Criteria for Antiosteoporosis Treatment After Simulated Elagolix Treatment

	All patients	Patients who met threshold for initiation of antiosteoporosis medication after elagolix treatment	
		150‐mg QD for 12 months	200‐mg BID for 3 months
*N*	2303	16	15
Age, years			
Mean (SD)	63 (8)	69 (8)	68 (8)
Median (min, max)	62 (50, 79)	72 (54, 79)	71 (54, 79)
Race/ethnicity, *n* (%)			
Black	507 (22.0%)	1 (6.3%)	1 (6.7%)
Hispanic	457 (19.8%)	4 (25.0%)	4 (26.7%)
White	1339 (58.1%)	11 (68.8%)	10 (66.7%)
BMI, kg/m^2^			
Mean (SD)	29.05 (5.99)	25.62 (3.05)	25.52 (3.12)
Median (min, max)	28.49 (13.18, 53.89)	26.33 (19.38, 32.57)	26.15 (19.38, 32.57)
BMD *T*‐score			
Mean (SD)	−1.0 (1.1)	−2.1 (0.6)	−2.2 (0.4)
Median (min, max)	−1.0 (−4.4, 5.0)	−2.2 (−2.8, −0.6)	−2.2 (−2.8, −1.5)
Non‐BMD FRAX factors, *n* (%)			
Prior fracture	84 (3.6%)	0 (0.0%)	0 (0.0%)
Parental history of fracture/osteoporosis	500 (21.7%)	5 (31.3%)	4 (26.7%)
Current smoking	386 (16.8%)	5 (31.3%)	5 (33.3%)
Glucocorticoid use	105 (4.6%)	0 (0.0%)	0 (0.0%)
Rheumatoid arthritis	216 (9.4%)	1 (6.3%)	1 (6.7%)
Alcohol use (2+ drinks/day)	159 (6.9%)	1 (6.3%)	1 (6.7%)

Patients met the threshold to initiate antiosteoporosis treatment if their 10‐year predicted risk of hip fracture was ≥3% or 10‐year probability of major osteoporotic fracture was ≥20%.

BID = Twice daily; QD = once daily.

### Impact of elagolix treatment on postmenopausal risk of fracture

The 10‐year risk of major osteoporotic fracture and risk of hip fracture were slightly greater among women aged 50 to 59 years with simulated prior elagolix treatment compared with women who were not treated with elagolix (Table 3). In women aged 50 to 59 years, the difference in median risk was 0.03% for major osteoporotic fracture and 0.01% for hip fracture in the 150‐mg QD group. For the older age strata, the differences between the elagolix‐treated and the control group were greater, although the increases in fracture risk based on the simulated elagolix treatment were still <0.9%, even among women at the highest risk (ages 70 to 79 years, BMD <25th percentile and FRAX score >90th percentile in the absence of elagolix).

**Table 3:** Impact of 150 mg Elagolix QD for 12 Months or 200 mg Elagolix BID for 3 Months on Median 10‐Year Risk of Hip Fracture and Major Osteoporotic Fracture: All Patients and by Percentile of Postmenopausal BMD (g/cm^2^) in the Absence of Elagolix Treatment

Age strata, years				Post‐menopausal BMD in the absence of elagolix treatment								
	All patients			< 25th percentile			25th to 75th percentile			>75th percentile		
	Not treated with elagolix	Treated with elagolix	Difference	Not treated with elagolix	Treated with elagolix	Difference	Not treated with elagolix	Treated with elagolix	Difference	Not treated with elagolix	Treated with elagolix	Difference
150 mg QD for 12 months												
Median 10‐year risk of hip fracture, %												
50–59	0.18	0.19	0.01	0.83	0.87	0.04	0.18	0.19	0.01	0.02	0.02	0.00
60–69	0.49	0.51	0.02	1.87	1.95	0.09	0.48	0.50	0.02	0.10	0.10	0.00
70–79	2.14	2.22	0.08	7.03	7.31	0.28	2.09	2.18	0.09	0.41	0.42	0.01
Median 10‐year risk of major osteoporotic fracture, %												
50–59	4.70	4.73	0.03	7.23	7.34	0.11	4.84	4.88	0.04	2.62	2.64	0.02
60–69	6.97	7.03	0.07	10.89	11.08	0.19	7.08	7.15	0.07	4.52	4.56	0.04
70–79	10.68	10.83	0.15	19.71	20.16	0.45	10.81	10.96	0.15	6.21	6.27	0.06
200 mg BID for 3 months												
Median 10‐year risk of hip fracture, %												
50–69	0.18	0.19	0.01	0.83	0.87	0.04	0.18	0.19	0.01	0.02	0.02	0.00
60–69	0.49	0.51	0.02	1.87	1.95	0.09	0.48	0.50	0.02	0.10	0.10	0.00
70–79	2.14	2.22	0.08	7.03	7.29	0.26	2.09	2.17	0.08	0.41	0.42	0.01
Median 10‐year risk of major osteoporotic fracture, %												
50–69	4.70	4.73	0.02	7.23	7.33	0.10	4.84	4.88	0.04	2.62	2.64	0.02
60–69	6.97	7.03	0.06	10.89	11.07	0.18	7.08	7.15	0.06	4.52	4.56	0.04
70–79	10.68	10.82	0.14	19.71	20.14	0.43	10.81	10.95	0.14	6.21	6.27	0.06

BID = Twice daily; QD = once daily.

The difference in risk of osteoporotic fracture or hip fracture between the elagolix‐treated group and the comparator group was similar in both elagolix‐dosing scenarios (Table 3). More specifically, the median risk of osteoporotic fracture caused by elagolix treatment was almost the same for both elagolix doses in younger age strata: 4.70% to 4.73% (ages 50 to 59 years) and 6.97% to 7.03% (ages 60 to 69 years). For the oldest age strata (ages 70 to 79 years), the median risk of osteoporotic fracture was 10.68% without elagolix, 10.82% for patients receiving 200‐mg BID for 3 months, and 10.83% for patients receiving 1 year of 150‐mg QD of simulated elagolix treatment. The increase in the median risk of hip fracture caused by elagolix treatment was similar for both dose and duration scenarios across all age strata: change from 0.18% to 0.19% (ages 50 to 59 years), from 0.49% to 0.51% (ages 60 to 69 years), and from 2.14% to 2.22% (ages 70 to 79 years).

Among patients who were 70 to 79 years old and received a simulated premenopausal dose of 150‐mg elagolix QD for 12 months, the NNH was 643 for one additional hip fracture and 454 for one additional major osteoporotic fracture. For the 200‐mg BID scenario, the NNH was 684 for hip fracture and 483 for major osteoporotic fracture (Table 4).

**Table 4:** Impact of 150 mg Elagolix QD for 12 Months or 200 mg Elagolix BID for 3 Months on the Mean 10‐Year Risk of Hip Fracture and Major Osteoporotic Fracture and NNH Analysis

Age strata, years	Mean 10‐year risk of fracture			
	Not treated with elagolix	Treated with elagolix	Difference	NNH
150 mg QD for 12 months				
50–59 (*N* = 830)				
Hip fracture	0.45%	0.48%	0.03%	3979
Osteoporotic fracture	5.87%	5.93%	0.06%	1632
60–69 (*N* = 860)				
Hip fracture	0.97%	1.01%	0.05%	2203
Osteoporotic fracture	8.40%	8.51%	0.11%	938
70–79 (N = 613)				
Hip fracture	4.34%	4.49%	0.16%	643
Osteoporotic fracture	12.63%	12.85%	0.22%	454
200 mg BID for 3 months				
50–59 (*N* = 830)				
Hip fracture	0.45%	0.48%	0.02%	4252
Osteoporotic fracture	5.87%	5.93%	0.06%	1738
60–69 (*N* = 860)				
Hip fracture	0.97%	1.01%	0.04%	2342
Osteoporotic fracture	8.40%	8.50%	0.10%	1000
70–79 (*N* = 613)				
Hip fracture	4.34%	4.48%	0.15%	684
Osteoporotic fracture	12.63%	12.84%	0.21%	483

BID = Twice daily; NNH = number needed to harm; QD = once daily.

### Impact of elagolix treatment on reaching the thresholds to initiate antiosteoporosis treatment

Overall, the proportion of patients reaching the risk‐based thresholds to initiate antiosteoporosis treatment increased between 0.23% to 1.79% with simulated elagolix treatment (Table 5). The effect generally increased with advancing age (mean age among patients who reached the threshold to initiate antiosteoporosis treatment because of elagolix treatment was 69 years old and 68 years old for the 150‐mg QD and 200‐mg BID groups, respectively, compared with 63 years old for the overall population), concurrent with decreasing BMD in the general referent population (BMD *T*‐score −2.09 in the 150‐mg QD group and −2.18 in the 200‐mg BID group, compared with −0.96 in the overall population; Table 2). The increase in the proportion of women estimated to reach the threshold to initiate antiosteoporosis treatment after elagolix 150‐mg QD for 12 months was 0.36% at age 50 to 59 years, 0.23% at age 60 to 69 years, and 1.79% at age 70 to 79 years. Patients treated with 150‐mg QD of elagolix for 1 year were observed to have a slightly higher increase in the proportion of patients reaching the thresholds to initiate antiosteoporosis treatment than the patients who received 200‐mg BID for 3 months in the 70‐ to 79‐years‐of‐age strata (1.79% versus 1.63%). The increase was the same for the two dosing scenarios in the 50‐ to 59‐years‐of‐age and 60‐ to 69‐years‐of‐age strata (0.36% and 0.23%, respectively; Table 5).

**Table 5:** Impact of 150‐mg Elagolix QD for 12 Months or 200‐mg Elagolix BID for 3 Months on Proportion of Patients Who Met Criteria for Antiosteoporosis Treatment: All Patients and by Percentile of Postmenopausal BMD in the Absence of Elagolix Treatment

Age strata, years	All patients			Post‐menopausal BMD in the absence of elagolix treatment								
				<25th percentile			25th to 75th percentile			>75th percentile		
	Not treated with elagolix	Treated with elagolix	Difference	Not treated with elagolix	Treated with elagolix	Difference	Not treated with elagolix	Treated with elagolix	Difference	Not treated with elagolix	Treated with elagolix	Difference
150 mg QD for 12 months												
Patients who met criteria for antiosteoporosis treatment, *N* (%)												
50–59	21 (2.53%)	24 (2.89%)	3 (0.36%)	17 (8.29%)	20 (9.76%)	3 (1.46%)	4 (0.96%)	4 (0.96%)	0 (0.00%)	0 (0.00%)	0 (0.00%)	0 (0.00%)
60–69	70 (8.14%)	72 (8.37%)	2 (0.23%)	56 (26.17%)	58 (27.1%)	2 (0.93%)	14 (3.24%)	14 (3.24%)	0 (0.00%)	0 (0.00%)	0 (0.00%)	0 (0.00%)
70–79	241 (39.31%)	252 (41.11%)	11 (1.79%)	139 (90.85%)	143 (93.46%)	4 (2.61%)	97 (31.6%)	103 (33.55%)	6 (1.95%)	5 (3.27%)	6 (3.92%)	1 (0.65%)
200 mg QD for 3 months												
Patients who met criteria for antiosteoporosis treatment, *N* (%)												
50–59	21 (2.53%)	24 (2.89%)	3 (0.36%)	17 (8.29%)	20 (9.76%)	3 (1.46%)	4 (0.96%)	4 (0.96%)	0 (0.00%)	0 (0.00%)	0 (0.00%)	0 (0.00%)
60–69	70 (8.14%)	72 (8.37%)	2 (0.23%)	56 (26.17%)	58 (27.1%)	2 (0.93%)	14 (3.24%)	14 (3.24%)	0 (0.00%)	0 (0.00%)	0 (0.00%)	0 (0.00%)
70–79	241 (39.31%)	251 (40.95%)	10 (1.63%)	139 (90.85%)	143 (93.46%)	4 (2.61%)	97 (31.6%)	103 (33.55%)	6 (1.95%)	5 (3.27%)	5 (3.27%)	0 (0.00%)

Patients met the threshold to initiate antiosteoporosis treatment if their 10‐year predicted risk of hip fracture was ≥3% or 10‐year probability of major osteoporotic fracture was ≥20%.

BID = Twice daily; QD = once daily.

### Subgroup analysis

The impact of elagolix treatment on fracture risk was greater among women with lower BMD (eg, postmenopausal BMD in the absence of elagolix treatment <25^th^ percentile; Table 3). Similarly, the impact of elagolix on reaching the thresholds for antiosteoporosis treatment was greater for women with a lower BMD relative to other women (Table 5). However, the increase in the proportion of women who reached the treatment thresholds between the elagolix‐treated group and the control group was still less than 2.0% across all age strata and in both elagolix‐dosing scenarios. No additional patients with postmenopausal BMD above the 75th percentile required antiosteoporosis treatment as a result of elagolix treatment.

### Sensitivity analysis

A sensitivity analysis was conducted by modeling the effect on BMD on a population of patients in which 50% were treated with 150‐mg QD elagolix for 12 months and 50% were treated with 200‐mg BID elagolix for 3 months. The results were similar as the dosing scenarios presented here (data not shown). The proportion of NHANES patients with rheumatoid arthritis exceeded the national prevalence; thus, another sensitivity analysis was conducted by assuming that none of the patients had rheumatoid arthritis. Although the long‐term risks of fracture were systematically smaller in this scenario, the differences between the groups treated or not treated with elagolix remained the same (data not shown).

## Discussion

The present study used clinical trial data from the elagolix clinical development program to quantify the on‐treatment BMD reduction and after‐treatment reduction, and then used a large real‐world data source (NHANES) to simulate expected changes in long‐term fracture risk and in the proportion of patients reaching risk‐based thresholds to initiate antiosteoporosis treatment in premenopausal women treated with elagolix for moderate to severe endometriosis pain. NHANES data were used in the simulations because the NHANES performed DXA measurements on women with a wide range of ages, regardless of risk factors or indications, which allowed age‐group‐stratified analyses to demonstrate the impact of elagolix on long‐term fracture risks by age. Additionally, the observed postmenopausal BMD in the NHANES data reflected the impact of factors other than elagolix on BMD over years. The simulations provided quantitative evidence that although the impact of elagolix on the 10‐year risk of major osteoporotic fracture or hip fracture increases with age, the average impact of elagolix following menopause is small. The larger effects of elagolix in older women reflects greater baseline FRAX scores attributable to age, resulting in larger absolute increases for the same BMD reduction and making it easier to reach the antiosteoporosis treatment threshold. Further, this study found that exposure to elagolix during premenopause in the defined dosing and duration scenarios here, should have a minimal effect for most women on the likelihood of meeting risk‐based treatment guidelines. Finally, the impact appeared to be similar in the exposure to 150‐mg QD regimen for 12 months and the exposure to 200‐mg BID for 3 months.

These results have important clinical implications that can be interpreted in several ways. First, the absolute change in risk of postmenopausal fractures caused by elagolix exposure was low, as evidenced by an NNH of over 450 for major osteoporotic fractures and over 600 for hip fractures. For comparison, the NNH for renal insufficiency in patients undergoing intensive blood pressure control is 50.^(^ ^30^ ^)^ With respect to statin use over 5 years, the NNH was 125 to 250 for diabetes mellitus.^(^ ^31^ ^)^ Additionally, in women with low BMD, osteoporotic medications, such as bisphosphonates, can mitigate this risk as they can reduce fracture risk by 40% to 70%.^(^ ^32^ ^)^ The results indicate that elagolix has minimal impact on long‐term bone health and outcomes, which can help guide clinical decision‐making regarding elagolix treatment for endometriosis patients. Second, the impact of elagolix exposure in premenopausal women can be interpreted as how many years sooner would a woman need to initiate antiosteoporosis medication because of premenopausal bone loss. The average femoral neck BMD for women aged 70 to 79 years is 0.664 g/cm^2^ (Hologic units). With that BMD, using FRAX, the average woman reaches the 3% 10‐year hip‐fracture‐treatment threshold between 77 and 78 years of age. If a woman's BMD is 3% lower (BMD of 0.644 g/cm^2^) based on premenopausal elagolix use, the treatment threshold is reached at age 75 to 76 years. If a woman's BMD is 6% lower (BMD of 0.624 g/cm^2^), the treatment threshold is reached at age 74 to 75 years. Therefore, if BMD in older ages is 6% lower because of premenopausal exposure to elagolix, this would result in the need for antiosteoporosis medication 2 to 4 years sooner.

Results from an analysis by Binkley et al. provide a framework to aid this assessment.^(^ ^23^ ^)^ Their simulation indicated that women with a peak bone mass above the population median could withstand up to a 10% decrease in BMD without altering the likelihood of reaching thresholds for the initiation of antiosteoporosis treatment, whereas women in the 25th percentile could tolerate up to a 4% loss. Similar to the report by Binkley et al., the present study used data from NHANES as well as FRAX, a previously validated tool used to predict long‐term risks of osteoporotic and hip fractures. However, instead of assuming varying degrees of drug‐induced BMD losses as in Binkley et al., the present study was able to apply actual trial‐derived estimates from the elagolix development program to model on‐treatment BMD reduction as well as posttreatment recovery. Unlike the present study, Binkley et al. additionally used the trabecular bone score (TBS) to assess fracture risk. Although BMD is traditionally used to assess fracture risk, TBS is a measure of bone texture from spine DXA images that can be used as a BMD‐independent adjustment factor for FRAX to further classify fracture risks.^(^ ^33^, ^34^ ^)^ Unfortunately, the role of TBS cannot be assessed in the present study as it was not available from the trial data.

The impact of elagolix on long‐term bone outcomes is important to understand for informed benefit–risk decision‐making. The time between elagolix exposure in the premenopausal period and the beginning of the risk window for postmenopausal fracture could average three or more decades, making direct evaluation of this safety question infeasible. Here, using data from the pivotal development program and applying estimates to a nationally representative population from NHANES, this simulation found relatively minimal impact of treatment, under the studied scenarios, on long‐term fracture risk or time to meeting risk‐based treatment targets in the overall endometriosis population (as reflected by the NNH estimates). This is a population‐level study and should not be interpreted as an individual risk‐prediction tool.

### Limitations

The findings of this study should be interpreted within the context of certain limitations. First, because of the lack of real‐world data on postmenopausal BMD and fractures among women with prior elagolix treatment, simulated data based on trial‐derived estimates, NHANES data, and the FRAX algorithm were used in this study. The clinical trial data may not capture all potential effect modifiers of elagolix on BMD and did not record non‐BMD measures of bone quality and microarchitecture (eg, TBS). The factors that were not selected by the trial‐derived models based on model fit may impact the BMD changes. In addition, factors not included in the FRAX algorithm (eg, the use of pain control medications) may also contribute to long‐term risk of fractures, which were not considered in the study. Second, although the approved duration of elagolix treatment is up to 24 months, the clinical trial experience included only 12 months of treatment duration; therefore, the effects of longer‐term treatment based on observed data could not be quantified. Similarly, the effects of recovery were also limited by the availability of follow‐up data from the trials. Third, the models estimating the treatment effect of elagolix and the recovery effect aimed to capture the overall trend in BMD changes on an aggregate level, rather than predicting changes in BMD for individual patients. Fourth, the mean values reported in this study do not account for the distribution of starting BMD and/or the distribution of the loss and recovery of BMD. In addition, certain assumptions have been made in the treatment simulation and the risk calculation. For example, one‐half of the FDA‐approved elagolix treatment duration was used to account for the variety in treatment duration in real‐world practice; consumption of two or more units of alcohol per day in the NHANES data was used to approximate the risk factor “consumption of three or more units of alcohol per day” in the FRAX tool. Finally, because of limitations in the data available in the NHANES database, thresholds for assessment of recommendations to initiate antiosteoporosis treatment were based solely on 10‐year risks of osteoporotic and hip fracture, whereas the guideline recommendations from the National Osteoporosis Foundation are based on additional criteria, including history of hip or vertebral fracture and *T*‐score at the femoral neck, total hip, or lumbar spine. This may limit the generalizability of results concerning recommendation for antiosteoporosis treatment.

## Conclusions

Although there is a risk of bone loss with elagolix, the impact of elagolix on the long‐term risk of fracture and on the likelihood to meet risk‐based treatment thresholds was minimal and was similar between the two approved elagolix dose and duration scenarios. The quantification of the long‐term consequences of elagolix related premenopausal BMD loss provides clinicians and other stakeholders with additional quantitative information about the risk–benefit profile of this endometriosis treatment.

## Disclosures

RDK, SEC, LRW, AMS, and MCS are employees of AbbVie receiving stock and/or stock options. WG, HY, SK, JZ, and CX are employees of Analysis Group, Inc, which has received consulting fees from AbbVie. NBW is a paid consultant to Abbvie but did not receive any payment paid consultant to Abbvie but did not receive any payment for contributions to this manuscript or authorship. WDL has no conflicts to disclose. No authors received payment for authorship.

## Author contributions

**Ryan Kilpatrick:** Conceptualization; formal analysis; investigation; methodology; resources; supervision; writing‐original draft; writing‐review and editing. **William Leslie:** Conceptualization; investigation; methodology; writing‐review and editing. **Lani Wegrzyn:** Conceptualization; investigation; methodology; writing‐review and editing. **Wei Gao:** Conceptualization; formal analysis; investigation; methodology; project administration; supervision; validation; writing‐original draft; writing‐review and editing. **Hongbo Yang:** Conceptualization; formal analysis; investigation; methodology; project administration; supervision; validation; writing‐original draft; writing‐review and editing. **Ahmed Soliman:** Investigation; writing‐review and editing. **Sarah Koenigsberg:** Formal analysis; investigation; methodology; validation; writing‐original draft; writing‐review and editing. **Jia Zhong:** Formal analysis; investigation; methodology; validation; writing‐original draft; writing‐review and editing. **Cheryl Xiang:** Formal analysis; investigation; methodology; validation; writing‐original draft; writing‐review and editing. **Nelson Watts:** Conceptualization; investigation; writing‐original draft; writing‐review and editing.

Authors’ roles: Study design: all authors. Data analysis: WG, HY, SK, JZ. Data interpretation: all authors. Manuscript draft and revisions: all authors. All authors take full responsibility for the integrity of the data analysis.

## Peer Review

The peer review history for this article is available at https://publons.com/publon/10.1002/jbm4.10401.
